# Investigating the cause of increased tetracycline-resistant *Neisseria gonorrhoeae* in England, 2016–20

**DOI:** 10.1093/jac/dkae073

**Published:** 2024-03-22

**Authors:** Rachel Pitt-Kendall, Suzy Sun, Stephen Hughes, Rachel Merrick, Hugo Donaldson, Michael Rayment, Zdravko Ivanov, Michaela Day, Aisha Bari, Monica Rebec, Emma Callan, Hamish Mohammed, Katy Sinka, Michelle Cole, Helen Fifer

**Affiliations:** UK Health Security Agency, London, UK; UK Health Security Agency, London, UK; Chelsea & Westminster NHS Foundation Trust, London, UK; UK Health Security Agency, London, UK; Chelsea & Westminster NHS Foundation Trust, London, UK; Chelsea & Westminster NHS Foundation Trust, London, UK; Imperial College London, London, UK; UK Health Security Agency, London, UK; UK Health Security Agency, London, UK; North West London Pathology, London, UK; North West London Pathology, London, UK; UK Health Security Agency, London, UK; UK Health Security Agency, London, UK; UK Health Security Agency, London, UK; UK Health Security Agency, London, UK; UK Health Security Agency, London, UK

## Abstract

**Background:**

Antimicrobial resistance in *Neisseria gonorrhoeae* is a global public health concern. Tetracycline resistance (TetR) increased from 39.4% to 75.2% between 2016 and 2021 in *N. gonorrhoeae* isolates collected through national surveillance in England, despite the absence of use of tetracyclines for the treatment of gonorrhoea.

**Objectives:**

We investigated whether there was correlation between bacterial sexually transmitted infection (STI) tests performed and treatment with antimicrobials, with increased TetR in *N. gonorrhoeae*.

**Methods:**

We examined correlations between bacterial STI tests, antimicrobial treatment and TetR in *N. gonorrhoeae*, using national surveillance data from three large sexual health services (SHS) in London during 2016–20. Doxycycline prescribing data and antibiograms of a non-STI pathogen from distinct patient groups (sexual health, obstetric and paediatric), at a large London hospital, were analysed to identify if doxycycline use in SHS was associated with resistance in a non-STI organism.

**Results:**

A substantial increase in TetR was observed, particularly in isolates from gay, bisexual and other MSM (GBMSM). Strong positive correlations were observed exclusively in GBMSM between *N. gonorrhoeae* TetR and both bacterial STI tests (*r *= 0.97, *P *= 0.01) and antimicrobial treatment (*r *= 0.87, *P *= 0.05). Doxycycline prescribing increased dramatically during the study period in SHS. Prevalence of TetR in *Staphylococcus aureus* was higher in isolates sourced from SHS attendees than those from other settings.

**Conclusions:**

Frequent screening of GBMSM at higher risk of STIs, such as those on pre-exposure prophylaxis (PrEP) leading to/and increased use of doxycycline for the treatment of diagnosed infections, may account for the increase in TetR in *N. gonorrhoeae*.

## Introduction

*Neisseria gonorrhoeae* is the second most commonly reported sexually transmitted infection (STI) in England with 82 595 diagnoses in 2022.^1^ This obligate human pathogen can infect the urogenital tract, rectal mucosa and the pharynx. Symptoms vary depending on the site of infection, but *N. gonorrhoeae* can cause urethritis and cervicitis, with complications including pelvic inflammatory disease (PID) and infertility. Whilst the majority of urogenital infections in men are symptomatic (>90%), a large proportion of women may be asymptomatic at this site.^2^ Extra-genital infections are most commonly detected in gay, bisexual and other MSM (GBMSM) and are usually asymptomatic. This is thought in part likely due to the recommendation for routine screening at extra-genital sites for GBMSM.^3^ Diagnoses in GBMSM accounted for 47.1% of overall *N. gonorrhoeae* diagnoses in England in 2022, indicating a significant burden of infection within this group.^4^

*N. gonorrhoeae* has been a WHO priority organism since 2012^5^ due to increasing antimicrobial resistance (AMR). AMR in this organism was first reported in the 1970s in female sex workers in East Asia.^6^ Since then, AMR has repeatedly emerged in GBMSM, sometimes years ahead of emergence in heterosexual populations.^6,7^ The Gonococcal Resistance to Antimicrobials Surveillance Programme (GRASP) reports antimicrobial susceptibility data for consecutive isolates of *N. gonorrhoeae* collected over a 2 or 3 month period each year (length of collection period dependent on diagnosis rates) at collaborating laboratories across England and Wales. Antimicrobial susceptibility testing (AST) of isolates, against current and historical recommended treatment options (specifically ceftriaxone, cefixime, azithromycin, tetracycline, penicillin, ciprofloxacin, gentamicin and spectinomycin), is performed at the national reference laboratory at the UK Health Security Agency (UKHSA) via the agar dilution method.^8^ Antibiograms are linked to enhanced demographic, behavioural and patient-specific data to give an in-depth view of the epidemiology of AMR in *N. gonorrhoeae.*^8^

Tetracyclines are a broad-spectrum, bacteriostatic class of antimicrobials, which are used to treat many infections of the skin and soft tissues, respiratory tract and genital tract. Tetracyclines act by binding to the 30S ribosomal subunit, preventing binding of aminoacyl-tRNA to the mRNA–ribosomal complex, thus inhibiting protein synthesis.^9^ Their use is contraindicated during pregnancy due to potential effects on skeletal development and later discolouration of the child’s teeth.^10^ Paediatric (<12 years old) treatment with tetracycline should be cautioned due to the latter reason.^10^ Tetracycline was historically used as a treatment for *N. gonorrhoeae*, particularly in patients with penicillin allergies. However, emergence of chromosomal^11^ then plasmid-mediated resistance^12^ in the 1950s and 1980s, respectively, resulted in its removal from treatment guidelines worldwide. Low-level tetracycline resistance (MIC of >1.0 but ≤8.0 mg/L) results from mutations in several chromosomal *N. gonorrhoeae* genes causing (i) decreased affinity of tetracycline for its target (i.e. modification of the S10 ribosomal protein encoded by the *rpsJ* gene, which forms part of the 30S ribosomal subunit), (ii) decreased influx of tetracycline into the cell through the PorB porin and (iii) increased efflux of the antimicrobial through the MtrCDE efflux pump.^2^ Plasmid-mediated tetracycline resistance resulting in high-level resistance (MIC ≥ 16 mg/L)^12,13^ is due to a *tet*(M) resistance gene insert in *N. gonorrhoeae* conjugative plasmids.^14,15^

There has been increasing concern that the intensive use of antimicrobials for the treatment of STIs may be driving AMR both in the specific ‘target’ organism being treated and in other organisms present in the patient; the so-called ‘bystander effect’.^16,17^ This is of particular concern in populations that are exposed to repeated treatment, such as GBMSM with multiple new or casual partners, as they are recommended to screen for STIs every 3 months^18^ and may therefore be diagnosed with bacterial STIs and prescribed antibiotics more regularly than heterosexuals.^19,20^ Although doxycycline (a tetracycline) is not used to treat gonorrhoea, it is used to treat non-specific urethritis, as well as common STIs such as chlamydia and *Mycoplasma genitalium*.^21,22^ Since September 2018 the British Association for Sexual Health and HIV (BASHH) have preferentially recommended a 7 day course of doxycycline over 1 g azithromycin for the treatment of uncomplicated chlamydia infections due to concerns of selection pressure and macrolide resistance in *M. genitalium*.^23^ Lymphogranuloma venereum (LGV), the invasive form of *Chlamydia trachomatis* reported almost exclusively in GBMSM, is treated with a 21 day course of twice-daily 100 mg doxycycline.^21^ Additionally, use of doxycycline as post-exposure prophylaxis (dPEP) by GBMSM may be increasing.^24^ Between 2016 and 2021 the prevalence of tetracycline resistance (MIC > 1 mg/L) in *N. gonorrhoeae* isolates tested through GRASP increased from 39.4% to 75.2%, with the most marked increase amongst GBMSM (41.6% in 2015 to 78.2% in 2021).^25,26^

We hypothesized that the increasing rates of tetracycline resistance in *N. gonorrhoeae* may be due to bystander effect and sought to investigate this further through ecological analysis.

## Methods

### Data sources

Data were obtained from GRASP and the GUMCAD STI surveillance system^27^ (the national STI surveillance system in England). Detailed methodologies and protocols for both GRASP and GUMCAD have previously been published.^8,28^ Data on antimicrobial prescribing at Chelsea and Westminster NHS Trust were extracted from REFINE (Rx Info Ltd, UK). Chelsea and Westminster NHS Trust was selected to participate in this analysis as this centre contributes the largest number of isolates to GRASP.

Data on sexual health service provision for Chelsea and Westminster sexual health services (SHSs) (56 Dean Street, 10 Hammersmith Broadway and John Hunter Clinic) between 2015 and 2019 were extracted from the GUMCAD STI surveillance system. These data were used to calculate the number of episodes of care with at least one bacterial STI test (chlamydia, gonorrhoea, syphilis or *M. genitalium*) and episodes of care for which an antimicrobial was likely to have been prescribed for treatment. As antimicrobial prescribing is not recorded in GUMCAD, treatment with antimicrobials was assumed using a composite measure of (i) being diagnosed with a bacterial STI (chlamydia, gonorrhoea, syphilis, LGV, *M. genitalium*) or non-specific genital infection OR (ii) being a contact of someone diagnosed with chlamydia, gonorrhoea, syphilis, non-specific genital infection, PID or *M. genitalium*. An episode of care included all consultations within a 42 day period. To avoid double-counting, only one test, diagnosis (or presumptive treatment) respectively was counted within a 42 day episode of care. Therefore, episodes of care with a bacterial STI test and episodes with antimicrobial treatment are a subset of the total number of episodes of care; however, those treated with antimicrobials are not wholly a subset of those with a bacterial STI test. Tetracycline susceptibility data for *N. gonorrhoeae* isolates cultured from individuals attending a Chelsea and Westminster SHS were obtained from GRASP for the period 2016 to 2020. Data from GUMCAD and GRASP were not restricted to the same set of individuals and thus data on sexual health promotion and tetracycline susceptibility depict different cohorts.

### Ecological analysis

Pearson’s correlation test^7^ was used to measure correlations between the annual number of episodes of care with at least one bacterial STI test and the annual number treated with antimicrobials (described in Table 1) against the proportion of tetracycline-resistant (MIC > 1 mg/L, EUCAST breakpoint at the time the isolates were collected) *N. gonorrhoeae* in the following year. To calculate tetracycline resistance, the proportion of tetracycline-resistant *N. gonorrhoeae* isolates in GRASP for the Chelsea and Westminster clinics (relative to the total number of *N. gonorrhoeae* isolates in GRASP for those clinics) was determined. A 1 year lag was used when performing this analysis between antimicrobial consumption and detection of AMR, as this was shown to best illustrate the emergence of penicillin and macrolide resistance in *Streptococcus pneumoniae*.^29,30^

**Table 1.** Chelsea and Westminster SHS episodes of care as reported through GUMCAD and tetracycline-resistant (Tet-R)^a^  *N. gonorrhoeae* in GRASP between 2015 and 2020^b^, stratified by sexual risk

Sexual risk	Year	Episodes of care^c^	Bacterial STI test^d^	Treated with antimicrobials^e^	Isolates tested (*n*)	Low-level Tet-R	High-level Tet-R	Total Tet-R	Low-level Tet-R, % (95% CI)	High-level Tet-R, % (95% CI)	Total Tet-R, % (95% CI)
Heterosexual men and all women	2015	**75 655**	**61 381**	**5708**	N/A	N/A	N/A	N/A	N/A	N/A	N/A
	2016	80 821	65 980	5729	47	12	6	18	25.5 (13.2–44.6)	12.8 (4.7–27.8)	**38.3** (**22.7–60.5)**
	2017	80 466	65 500	5933	33	7	9	16	21.2 (8.5–43.7)	27.3 (12.5–51.8)	48.5 (27.7–78.7)
	2018	69 563	54 355	6532	50	11	12	23	22 (11.0–39.4)	24 (12.4–41.9)	46 (29.1–69.6)
	2019	**57 644**	**42 558**	**6507**	78	25	16	41	32.1 (20.7–47.3)	20.5 (11.7–33.3)	52.6 (37.7–71.3)
	2020	N/A	N/A	N/A	62	23	15	38	37.1 (23.5–55.7)	24.2 (13.5–39.9)	**61.3** (**43.4–84.1)**
GBMSM	2015	**59 326**	**49 540**	**14 479**	N/A	N/A	N/A	N/A	N/A	N/A	N/A
	2016	60 481	52 034	13 403	525	131	93	224	25 (20.9–29.6)	17.7 (14.3–21.7)	**42.7** (**37.3–48.6)**
	2017	68 275	58 448	15 280	452	113	141	254	25 (20.6–30.1)	31.2 (26.3–36.8)	56.2 (49.5–63.5)
	2018	67 430	60 021	19 410	377	148	101	249	39.3 (33.2–46.1)	26.8 (21.8–32.6)	66 (58.1–74.8)
	2019	**69 188**	**61 207**	**22 575**	494	212	163	375	42.9 (37.3–49.1)	33 (28.1–38.5)	75.9 (68.4–84.2)
	2020	N/A	N/A	N/A	430	295	64	359	68.6 (61.0–76.9)	14.9 (11.4–19.6)	**83.5** (**75.1–92.6)**
All^f^	2015	138 208	113 240	20 401	N/A	N/A	N/A	N/A	N/A	N/A	N/A
	2016	145 102	120 833	19 405	583	144	102	246	24.7 (20.8–29.1)	17.5 (14.3–21.2)	**42.2** (**37.1–47.8)**
	2017	152 264	126 580	21 505	502	124	156	280	24.7 (20.5–29.5)	31.1 (26.4–36.4)	55.8 (49.4–62.7)
	2018	140 050	116 551	26 280	492	184	134	318	37.4 (32.2–43.2)	27.2 (22.8–32.3)	64.6 (57.7–72.1)
	2019	132 958	108 280	30 079	580	240	183	423	41.4 (36.3–47.1)	31.6 (27.1–36.5)	72.9 (66.1–85.2)
	2020	N/A	N/A	N/A	519	335	80	415	64.5 (57.8–71.8)	15.4 (12.2–19.2)	**80** (**72.4–88.5)**

‘Episodes of care’, ‘bacterial STI test’ and ‘treated with antibiotics’ are independent variables, not subsets of each other.

^a^Tetracycline resistance is defined as MIC > 1 mg/L (EUCAST breakpoint at time of analysis) and stratified by low-level tetracycline resistance (MIC = 2 to 8 mg/L) and high-level tetracycline resistance (MIC > 8 mg/L).

^b^Independent variables (e.g. bacterial STI tests, antimicrobials prescribed) compared with dependent variable (proportion of gonococcal isolates resistant to tetracycline) in the following year, e.g. bacterial STI tests in 2015 compared with % resistant in 2016.

^c^An episode of care comprises one or more attendances within a 42 day period.

^d^Episode of care during which at least one test for chlamydia, gonorrhoea, syphilis and/or *M. genitalium* was carried out. To avoid double-counting, only one test is counted within a 42 day episode of care.

^e^Episode of care during which antimicrobial(s) were assumed to be prescribed. Derived as a composite measure of (i) being diagnosed with a bacterial STI (chlamydia, gonorrhoea, syphilis, LGV, *M. genitalium*) or non-specific genital infection OR (ii) being a contact of someone diagnosed with one of the following: chlamydia, gonorrhoea, syphilis, non-specific genital infection, PID, *M. genitalium.* To avoid double-counting, only one diagnosis or treatment respectively is counted within a 42 day episode of care.

^f^Includes those reported with an unspecified gender or sexual orientation.

### Doxycycline usage at Chelsea and Westminster NHS Trust

Doxycycline daily doses, in DDDs, dispensed at Chelsea and Westminster SHSs were analysed between April 2011 and March 2021. As a comparator, doxycycline daily doses dispensed in two non-SHS settings across the Trust in the same time period were analysed. Data were extracted from pharmacy dispensing records using the REFINE system.

### Tetracycline susceptibility in a non-STI pathogen

To investigate whether doxycycline use in SHSs was associated with resistance in a non-STI pathogen, a comparison was made of the tetracycline antimicrobial susceptibility of MSSA isolates from three clinically distinct patient groups. *Staphylococcus aureus* was selected as the comparator pathogen as it likely affects patients attending SHS and non-sexual health services equally, and longitudinal tetracycline susceptibility testing data for this organism were available. Methicillin-resistant isolates were excluded as the screening programme for infection prevention and control is not undertaken in the SHS population, unlike the admitted obstetric and paediatric groups. Isolates were collected between January 2018 and July 2021. Group one comprised patients attending Chelsea and Westminster SHSs where doxycycline exposure is common. Groups two and three comprised pregnant women under obstetric care and children (aged 1–8 years) treated by the Chelsea and Westminster paediatric team. As doxycycline use is not routinely prescribed in pregnancy and childhood, groups two and three were used as comparators. A single isolate was included for each patient in each group and where multiple were available, only the first was included. Erythromycin susceptibility was used as a comparator antibiotic. Macrolides can be administered to all patient groups. Chi-squared test was used to analyse prevalence of resistance to both antimicrobials in and outside of SHSs. AST was carried out by the Microbiology department at North West London Pathology, hosted by Imperial College Healthcare NHS Trust by disc diffusion using the EUCAST method; http://www.eucast.org. The laboratory is United Kingdom Accreditation Service (UKAS) accredited for this method and uses *S. aureus* ATCC 29213/National Collection of Type Cultures (NCTC) 12973 as the Quality Control strain.

A cumulative antibiogram was produced for each patient group.

### Statistical methods

All analyses pertaining to the Pearson’s correlation test were carried out using Stata 15 v.1.0 (StataCorp LP, College Station, TX, USA). Chi-squared test was used to analyse prevalence of resistance to tetracycline and erythromycin in and outside of Chelsea and Westminster SHSs.

### Ethics

In its role providing infectious disease surveillance, the UKHSA (formerly PHE) has approval to handle data obtained by the GUMCAD STI surveillance system and GRASP under Regulation 3 of the Health Service (Control of Patient Information) Regulations 2002.

Data from Chelsea and Westminster NHS Trust were handled securely by local clinical teams, who removed all patient identifiers and summarized the data, before providing to UKHSA. Ethical review was not sought for the use of these data locally in this manner, in line with HRA guidelines.

## Results

### Ecological analysis

A total of 708 582 episodes of care were provided by Chelsea and Westminster SHSs between 2015 and 2019. During this time, 585 484 episodes of care had at least one bacterial STI test (henceforth referred to as ‘bacterial STI tests’) and 117 670 episodes of care were estimated to have had at least one antimicrobial regimen prescribed for STI treatment (henceforth referred to as ‘treated with antimicrobials’). GBMSM accounted for 45.8% (324 700/708 582) of these episodes of care, 48.0% (281 250/585 484) of bacterial STI tests and 72.4% (85 147/117 670) of those treated with antimicrobials (Table 1).

**Figure 1.**
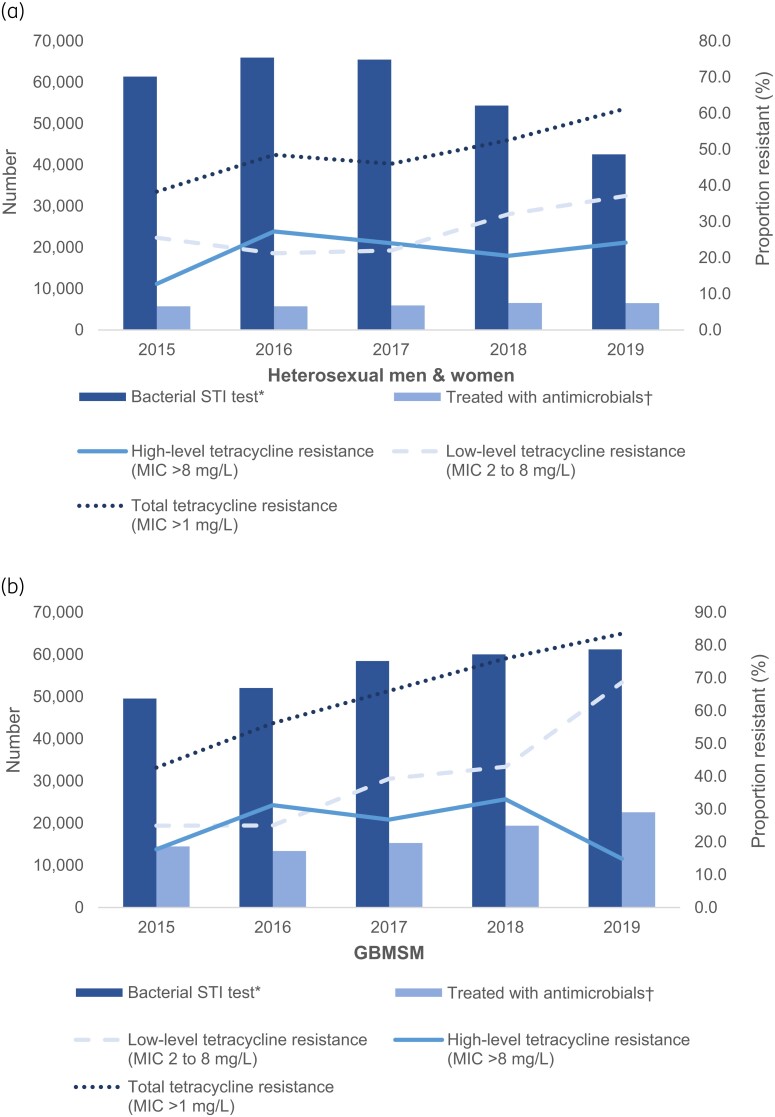

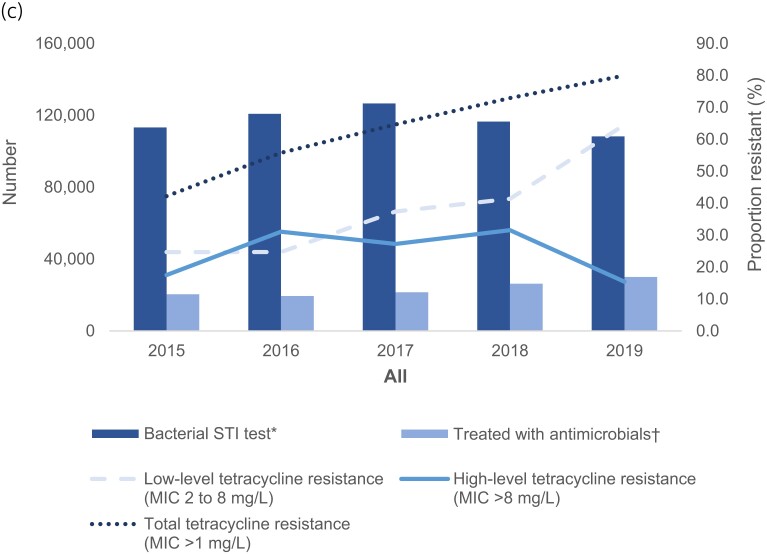
Trends in bacterial STI tests, treatment with antimicrobials and tetracycline-resistant *N. gonorrhoeae* at Chelsea and Westminster SHSs, between 2015 and 2019, by sexual risk (a–c). *Episode of care during which at least one test for chlamydia, gonorrhoea, syphilis and/or *M. genitalium* was carried out. To avoid double-counting, only one test is counted within a 42 day episode of care. †Episode of care during which antimicrobial(s) were prescribed. Derived as a composite measure of (i) being diagnosed with and treated with antimicrobials for a bacterial STI (chlamydia, gonorrhoea, syphilis, LGV, *M. genitalium*) or non-specific genital infection OR (ii) being given antimicrobial treatment as a contact of someone diagnosed with one of the following: chlamydia, gonorrhoea, syphilis, non-specific genital infection, PID, *M. genitalium*. To avoid double-counting, only one diagnosis or treatment respectively is counted within a 42 day episode of care. Tetracycline resistance is defined as an MIC > 1 mg/L (EUCAST breakpoint) and stratified by low-level tetracycline resistance (MIC = 2 to 8 mg/L) and high-level tetracycline resistance (MIC > 8 mg/L). Independent variables (e.g. bacterial STI tests, antimicrobials prescribed) compared with dependent variable (proportion of gonococcal isolates resistant to tetracycline) in the following year, e.g. bacterial STI tests in 2015 compared with % resistant in 2016. This figure appears in colour in the online version of *JAC* and in black and white in the print version of *JAC*.

Tetracycline resistance in *N. gonorrhoeae* increased steadily between 2016 and 2020 (from 42.2% to 80.0%, highlighted in Table 1) with a marked increase among GBMSM compared with heterosexual men and all women (40.8% versus 23.0% absolute increase in resistance between 2016 and 2020). The increase was largely driven by an increase in low-level tetracycline resistance whereas high-level tetracycline resistance fluctuated over this period (Table 1, Figure 1a–c).

Among heterosexual men and women, the number of episodes of care and bacterial STI tests decreased by 23.8% (75 655 versus 57 644) and 30.7% (61 381 versus 42 558) between 2015 and 2019, respectively (highlighted in Table 1, Figure 1a). Conversely, the number treated with antimicrobials increased by 14.0% (5708 in 2015 versus 6507 in 2019, highlighted in Table 1), primarily due to a 36.8% increase in partner notification (data not shown) while the number of bacterial STI diagnoses remained relatively unchanged. Tetracycline resistance in *N. gonorrhoeae* isolates from heterosexual men and women increased from 38.3% in 2016 to 61.3% in 2020 (highlighted in Table 1, Figure 1a).

Among GBMSM, the number of episodes of care increased by 16.6% between 2015 and 2019 (59 326 versus 69 188, highlighted in Table 1). Similarly, bacterial STI tests increased by 23.6% (49 540 in 2015 versus 61 207 in 2019, highlighted in Table 1). The number treated with antimicrobials increased by 55.9% (14 479 in 2015 versus 22 575 in 2019, highlighted in Table 1) driven by both a 48.4% rise in bacterial STI diagnoses and 36.8% rise in attendance as a result of partner notification (data not shown). Tetracycline resistance in *N. gonorrhoeae* isolates from GBMSM showed a 40.8% increase from 42.7% in 2016 to 83.5% in 2020 (highlighted in Table 1, Figure 1b).

Between 2016 and 2019 the dominant resistance mechanism in all patients was low-level chromosomally mediated resistance (MIC = 2–8 mg/L), comprising 56.7%–58.5% of overall tetracycline resistance, with the exception of 2017, during which high-level plasmid-mediated resistance (MIC > 8 mg/L) was the dominant mechanism. In 2020, a large shift towards low-level resistance was observed, whereby 80.7% of resistant isolates were low-level resistant isolates and appeared to have been driven by changes among GBMSM (82.2% were low-level versus 60.5% among heterosexual men and women).

### Pearson’s correlation

Using Pearson’s correlation test, strong positive correlations were observed between tetracycline resistance in *N. gonorrhoeae* and both bacterial STI tests (*r *= 0.97, *P* value = 0.01) and treatment with antimicrobials (*r *= 0.87, *P* value = 0.05) in GBMSM (Table 2). Treatment with antimicrobials was also shown to have weak evidence of correlation with tetracycline resistance among heterosexual men and women. No other correlations were observed, either for GBMSM or heterosexual men and women.

**Table 2.** Pearson’s correlation between sexual risk category and independent variables (i.e. bacterial STI test, antimicrobials prescribed) with tetracycline-resistant *N. gonorrhoeae* between 2015 and 2019

	Tetracycline resistance (MIC > 1 mg/L)					
	Heterosexual men and all women (*n* = 136)		GBMSM (*n* = 1461)		All^a^ (*n* = 1597)	
	*r*	*P* value	*r*	*P* value	*r*	*P* value
Bacterial STI test	−0.80	0.10	**0.97**	**0**.**01**	−0.21	0.74
Treated with antimicrobials	0.83	0.08	**0.87**	**0**.**05**	0.86	0.06

*r*, Pearson’s correlation coefficient. Evidence of strong positive/negative correlation highlighted in bold.

^a^Includes only those with declared gender/sexual orientation.

### Doxycycline usage at Chelsea and Westminster NHS Trust

Analysis of antimicrobial prescribing data at Chelsea and Westminster showed a dramatic increase in doxycycline daily doses between 2011 and 2021, most noticeably so in SHSs. Over the 10 year period, doxycycline daily dose prescribing increased from 11 716 DDDs in quarter 2 (Q2) 2011 to a peak of 84 078 DDDs in Q3 2019 in SHSs (data not shown). As expected, during 2020 there was a significant drop in the daily doses prescribed due to SHS access limitations as a result of restrictions introduced as part of the COVID-19 pandemic response. Doxycycline prescribing outside of SHSs also increased from 10 115 DDDs in Q2 2011 to a peak of approximately 18 353 DDDs in Q1 2020 (data not shown).

### Tetracycline susceptibility in S. aureus

Cumulative antibiograms for MSSA clinical isolates collected from each patient group are presented in Table 3. Rates of AST differed across settings. However, prevalence of resistance to both tetracycline and erythromycin was higher in MSSA clinical isolates collected from patients attending SHSs than those from other settings (sexual health versus non-sexual health; tetracycline: *P* ≤ 0.001, erythromycin: *P* ≤ 0.001; chi-squared test). Resistance within clinical isolates taken from an obstetric and paediatric population were used as comparisons due to the low rates of tetracycline prescribing expected in these two populations. Rates of tetracycline resistance were lower in both obstetric and paediatric populations compared with clinical isolates obtained from SHS patients.

**Table 3.** Cumulative antibiograms for tetracycline and erythromycin of MSSA isolates collected from patients attending sexual health care, obstetric or paediatric care

	SHS, *n* (%)	Obstetrics, *n* (%)	Paediatrics (1–8 years old), *n* (%)
Tetracycline	*N*= 377	(*N* = 99)	(*N* = 1714)
Susceptible (MIC ≤ 1 mg/L)	285 (75.6)	90 (90.9)	1589 (92.7)
Resistant (MIC > 2 mg/L)	92 (24.4)	9 (9.1)	125 (7.3)
Erythromycin	*N* = 345	*N* = 79	*N*= 1713
Susceptible (MIC ≤ 1 mg/L)	174 (50.4)	74 (93.7)	1379 (80.5)
Resistant (MIC > 2 mg/L)	171 (49.6)	5 (6.3)	334 (19.5)

## Discussion

Tetracycline resistance in *N. gonorrhoeae* increased from 39.4% to 62.9% between 2016 and 2020 in England (measured through isolates submitted to GRASP overall), and 42.2% to 80.0% in isolates submitted to GRASP from Chelsea and Westminster SHSs specifically. These observed increases occurred in the absence of use of doxycycline as a treatment for gonorrhoea. We investigated whether there was any correlation between bacterial STI tests performed and treatment with antimicrobials, with tetracycline resistance in *N. gonorrhoeae*.

During our study period (2015–19) the number of episodes of care at Chelsea and Westminster SHSs varied, with a decrease in attendances for heterosexual men and women and an increase for GBMSM. Bacterial STI tests reported by the Chelsea and Westminster SHSs decreased by 30.7% among heterosexual men and women and increased by 23.6% among GBMSM during the study period, which may be due to online sexual health providers upscaling testing, particularly in heterosexual men and women. However, estimated antimicrobial treatment increased in both groups, by 14.0% among heterosexual men and women and by 55.9% among GBMSM. This is perhaps not that surprising given that bacterial STI diagnoses are considerably higher among GBMSM.^4^

Testing in GBMSM increased by 12.3% between 2016 and 2017 (compared with 2%–5% increases in other years), which coincided with the launch of the pre-exposure prophylaxis (PrEP) HIV Impact Trial across England in October 2017^31,32^ and commencement of recommended quarterly STI screening for individuals taking PrEP.^18^ It has been suggested previously that intense screening, diagnosis and treatment of infections, such as that observed for individuals taking PrEP/GBMSM at high risk of STI infection, may temporarily reduce the prevalence of infections such as *N. gonorrhoeae* in the target population.^33^ Some sexual networks of GBMSM typically report high rates of partner change and partner concurrency.^34^ Within these networks, a prevalence equilibrium of *N. gonorrhoeae* and *C. trachomatis* of >10% is achieved.^35^

An unintended consequence of intense screening in dense networks may be increased selection pressure for development of AMR.^33^ Whilst tetracycline resistance increased for both sexual risk groups in our study, the most marked increase was observed in GBMSM (42.7% in 2016 to 83.5% in 2020). Indeed, positive correlations were seen between tetracycline-resistant *N. gonorrhoeae* and both bacterial STI tests performed (*r *= 0.97, *P* value = 0.01) and antimicrobial treatments prescribed (*r *= 0.87, *P* value = 0.05) in GBMSM only. Interestingly, during the study period the proportion of heterosexual men and women coinfected with *N. gonorrhoeae* and *C. trachomatis* increased by 4%, whilst the proportion of GBMSM coinfected increased by 9% (data not shown). However, there was no difference in the average proportion coinfected between the two risk groups (heterosexual men and women: 20% versus GBMSM: 21%) indicating that this was unlikely to be a contributing factor to differences in tetracycline resistance in *N. gonorrhoeae*. A possible explanation for the association between tetracycline-resistant *N. gonorrhoeae* and bacterial STI tests is that AMR emerges in a population exposed to heavy antimicrobial usage, and AMR is transmitted within this population rather than emerging *de novo* in the individual being treated.

However, treatment with antimicrobials appears to be a good predictor of tetracycline resistance across both groups. Interestingly, bacterial STI tests were negatively correlated with tetracycline resistance among heterosexual men and women, while the opposite was true among GBMSM. This may be reflective of different drivers for resistance between these groups, e.g. less frequent testing among heterosexual men and women may lead to more asymptomatic resistant infections circulating within sexual networks, while frequent testing among GBMSM leads to increased antimicrobial use. Consequently, bacterial STI tests may instead be a good proxy measure for belonging to a high-risk network rather than a predictor of AMR.

Use of doxycycline both inside and outside of sexual healthcare increased dramatically, at a large London hospital, over the last decade. Changes to management guidelines to promote narrow-spectrum antibacterials for gynaecological conditions, skin and soft tissue infections and adult lower respiratory tract infections account for the increase in use outside of the sexual health setting.^36,37^ Within sexual health, doxycycline is recommended as a first- or second-line therapy in 5 of 11 disease-specific guidelines.^21,38–41^ Further to this, doxycycline is recommended as a pre-treatment for *M. genitalium* infection prior to administration of an *M. genitalium*-specific therapy.^22^ In addition, approximately 1 in 10 GBMSM using HIV PrEP in an online community survey^42^ and 8% of people using PrEP attending a Chelsea and Westminster SHS specifically,^43^ reported self-sourcing STI prophylactic antimicrobials including doxycycline.^43^ More recently, a trial in the USA reported a reduction in gonorrhoea, chlamydia and syphilis in GBMSM taking dPEP within 72 h of condomless sex.^44^ Subsequently the first proposed national guideline for dPEP was presented for consultation by the CDC (USA) in October 2023.^45^ In England, the effectiveness of dPEP against gonorrhoea may be limited by the high prevalence of tetracycline resistance.

Previous studies looking at high background cephalosporin consumption (as defined as up to 7.2 daily doses/1000 population per day) in relation to AMR in *N. gonorrhoeae* have found evidence of bystander selection.^7,46^ Kenyon *et al.*^7^ reported that population-wide cephalosporin consumption could be used to predict the prevalence of reduced susceptibility or resistance of *N. gonorrhoeae* to extended-spectrum cephalosporins. Tedijanto *et al*.^46^ estimated that 28.6% of *N. gonorrhoeae* exposures to tetracyclines in the USA in 2011 were a result of prescription of the antimicrobial for treatment of another organism. In our study, prevalence of resistance to tetracycline in *S. aureus* was higher in isolates from the sexual health setting (*P  *≤* *0.001) than in obstetric or paediatric settings, which do not commonly use doxycycline treatment. Erythromycin resistance in *S. aureus* was also higher in isolates from SHSs, even though macrolides are prescribed to all patient groups analysed. This was not surprising given that until recently, azithromycin has been used extensively to treat STIs, and again reflects high antimicrobial exposure in high-risk networks. These data further support the theory that population-level antimicrobial exposure may inadvertently lead to bystander selection of resistance and highlights the importance of monitoring the impact of interventions such as dPEP on other non-STI organisms. However, it was not possible to demonstrate bystander selection conclusively in this study as specific prescribing data were unavailable.

Previously it has been reported that GBMSM with two to four gonorrhoea diagnoses in a 3 year period (used as a proxy for regular treatment) were more likely to harbour isolates with reduced susceptibility to cefixime and ceftriaxone.^47^ Other studies have found no association between azithromycin-resistant *N. gonorrhoeae* and prior exposure to azithromycin,^48,49^ possibly suggesting that increasing resistance is the result of population-wide antimicrobial use.

The analyses in this study are all ecological and are thus susceptible to the ecological fallacy. Trends at the individual level can therefore not be inferred from those observed at the population level. Our findings cannot, for example, be used to quantify the risk posed to an individual of acquiring infection with tetracycline-resistant *N. gonorrhoeae* following repeated SHS attendances or bacterial STI tests. It is also worth noting that although this analysis compares individuals in GUMCAD with individuals in GRASP in the following year, this was an ecological comparison of different cohorts. A further limitation of our analyses is that we did not adjust for confounding factors. While we observed associations between SHS attendance and bacterial STI tests among GBMSM with *N. gonorrhoeae* tetracycline resistance, Pearson’s correlation test does not prove causation and other, often complex, factors such as international travel and sexual exposure abroad likely influence the likelihood of resistance emerging in *N. gonorrhoeae*. Similarly, although we used a 1 year time lag to assess population-level behaviours (i.e. SHS attendance) with antimicrobial susceptibility as other authors have done,^6^ this lag has not been validated for describing changing trends in tetracycline resistance in *N. gonorrhoeae*.

To conclude, tetracycline resistance increased markedly in *N. gonorrhoeae* between 2016 and 2020, particularly in GBMSM, despite doxycycline not being used to treat gonorrhoea. We reported correlations between tetracycline-resistant *N. gonorrhoeae* and bacterial STI tests and antimicrobial treatment in GBMSM. The increase in tetracycline resistance in *N. gonorrhoeae* in GBMSM may be linked to increased STI testing frequency. Additionally, increased use of doxycycline for the treatment of other STIs and non-STI pathogens may contribute to tetracycline resistance in *N. gonorrhoeae* through bystander effect. These findings may have relevance to the development of guidelines on the use of antibiotic prophylaxis to prevent STIs.
